# Pott's Puffy Tumor: An Uncommon Clinical Entity

**DOI:** 10.1155/2012/386104

**Published:** 2012-10-04

**Authors:** Phillip T. Suwan, Suvarna Mogal, Subhash Chaudhary

**Affiliations:** ^1^Department of Surgery, Division of Otolaryngology, Southern Illinois University School of Medicine, P.O. Box 19638, Springfield, IL 62794-9638, USA; ^2^Department of Pediatrics, Southern Illinois University School of Medicine, Springfield, IL 62794-9638, USA

## Abstract

Although first described in 1760, Pott's puffy tumor is a lesser known clinical entity. Often seen as a complication of frontal sinusitis, Pott's is a frontal bone osteomyelitis with an associated subperiosteal abscess. Patients present with a fluctuant swelling of the scalp. The diagnosis is often confirmed with computed tomography (CT). Prompt surgical and medical treatments are the rule as there is the potential for significant morbidity if not quickly diagnosed and treated. Herein, we describe the presentation, diagnosis, and treatment of an 8-year-old female presenting with Pott's puffy tumor.

## 1. Introduction

Sir Percival Pott first described the phenomenon of a Pott's puffy tumor as frontal swelling with a subperiosteal abscess and extradural empyema in 1775 [1, 2]. Although rare since the advent of modern antibiotic therapy, one must have a high index of suspicion for this process given the risks of intracranial sequelae if left unaddressed. Herein, we describe a case of Pott's puffy tumor in an 8-year-old child with a seemingly indolent prehospital course.

## 2. Case Presentation

An 8-year-old female, with no significant past medical history, presented with swelling of her forehead and a low-grade fever. She had been in her usual state of health until 12 days prior to admission when she developed a fever (temperature: 38.8°C), headaches, nasal congestion, and a decreased energy level. She was observed and treated symptomatically. Eight days prior to admission, her fever persisted, and she developed swelling of the forehead which was more prominent in the glabellar area. She was started on Cefdinir 250 mg once daily (8 mg/kg/day) for the management of a presumed facial cellulitis. In addition, she was prescribed a nasal decongestant and systemic antihistamine therapy. Her headache and fever resolved; however, the swelling of the forehead progressed. Moreover, generalized swelling of the eyelids, cheeks, and face became obvious. She was admitted for further workup and management.

**Figure 1:**
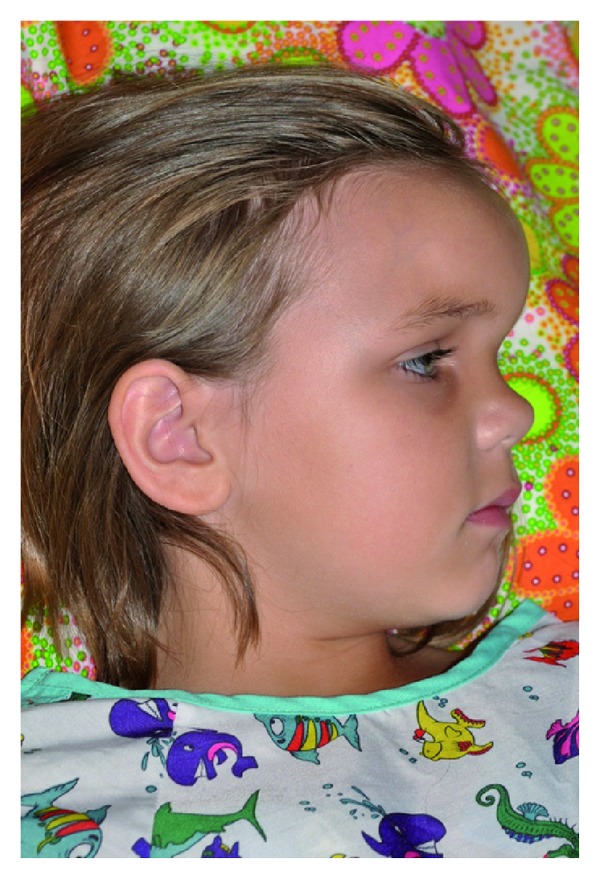
Sagittal view at the time of presentation demonstrating a notable amount of fullness over the area corresponding to the frontal sinus.

Physical examination on admission showed a well-nourished female in no acute distress. Her vital signs were unremarkable except for a temperature of 38.6°C. Her weight was at the 75th percentile (31.3 kg) and height was at the 50th percentile (133 cm). She answered questions appropriately and was cooperative with the examination. There was a pronounced, erythematous swelling of the forehead and edema of the eyelids (Figure 1). There were no sites of drainage or suggestions of trauma. Facial strength and sensation were intact. The nasal passages were widely patent. The turbinates were nonobstructive. The nasal mucosa was healthy, and no rhinorrhea was identified. Dentition was in good condition and without caries. The neck was soft, and no clinically significant lymphadenopathy was identified. Neurological examination was normal. The remainder of her physical exam was unremarkable.

Laboratory evaluations on the day of admission revealed the following: hemoglobin: 11.9 g/dL (reference range from, 11.5 to 15.5 g/dL); leukocytes: 17980/mm³ (reference range, 5000–15500/mm³) with absolute neutrophil count 13760/mm³ (reference range, 1500–9000/mm³); platelets: 411,000/mm³ (reference range, 150000–350000/mm³); erythrocyte sedimentation rate (ESR): 98 mm/hr (reference range, 0–20 mm/hr). 

**Figure 2:**
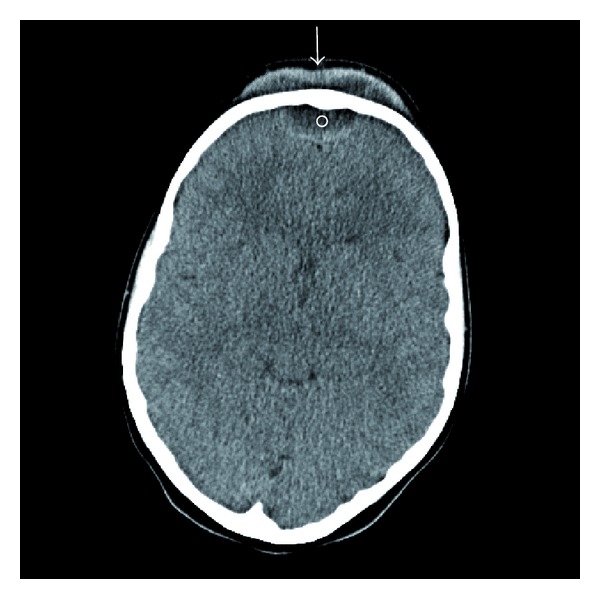
Axial CT of the brain demonstrating subperiosteal (arrow) and epidural abscesses (circle).

**Figure 3:**
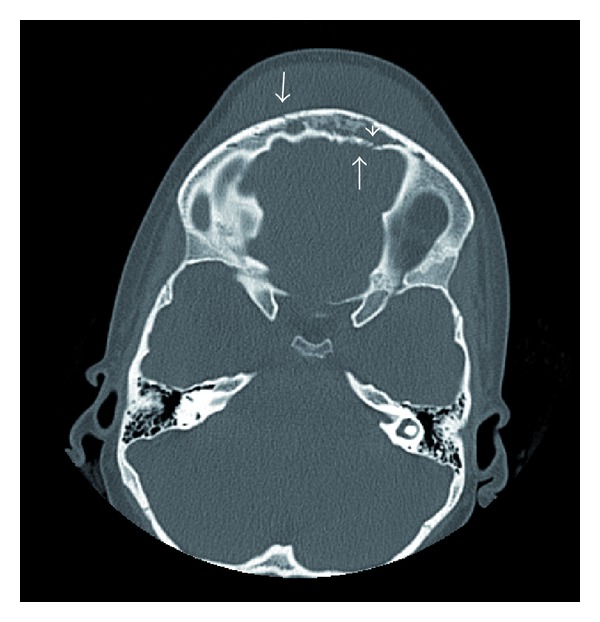
Axial CT without contrast on presentation demonstrating erosion of the anterior and posterior tables of the frontal sinus (arrows), frontal sinus opacification (arrowhead), and soft tissue swelling of the forehead.

Computerized axial tomography scans of the head and sinuses (Figures 2 and 3) revealed opacification of the ethmoid sinuses, the left maxillary sinus, and both frontal sinuses. There was erosion of the anterior and posterior tables of the frontal bone. There was soft tissue swelling with fluid attenuation measuring 6.9 × 1.4 cm—suggestive of a subperiosteal abscess. In addition, there was a 3.1 × 1.3 cm size epidural abscess in the frontal region of the brain.

After obtaining two blood cultures and a nasopharyngeal swab for bacterial cultures, the patient was started on high-dose intravenous antibiotic therapy with vancomycin, cefotaxime, and metronidazole. The otolaryngology and neurosurgery services were consulted, and surgical intervention was deemed necessary based on clinical exam and imaging studies. The following day she went to the operating room in a combined operation with otolaryngology and neurosurgery to address the forehead abscess, epidural abscess, and the underlying frontal sinus disease. An incision was made posterior to the hairline in the frontal region to approach the forehead abscess. This was incised, and 100 mL of frankly purulent fluid were expressed. A burr hole was created in the right frontal region, and again purulent fluid was identified. The epidural space was irrigated and suctioned. The dura was intact. Surgical drains were placed at the sites of the drained forehead and intracranial abscesses. To address the sinus disease, we utilized image guidance to create a left nasal antral window, bilateral frontal sinusotomies, anterior ethmoidectomies, and uncinectomies. Purulent fluid was obtained from the left middle meatus, left maxillary sinus, and left ethmoid bulla. Specimens were sent for gram stain, aerobic, anaerobic, fungal, and acid-fast cultures. 

Gram stain of the subgaleal and epidural purulent fluid showed many neutrophils, moderate gram-positive cocci in chains, and a few gram negative bacilli. Routine culture of both specimens grew *Streptococcus constellatus* susceptible to penicillin (MIC 0.032 *μ*g/mL by *E* test), cefotaxime (MIC 0.032 *μ*g/mL), and vancomycin (MIC < 1 *μ*g/mL). In addition, *Fusobacterium necrophorum* (susceptible to penicillin and metronidazole) was isolated on anaerobic culture of both specimens. Histologic examination of the sinus contents demonstrated mild, chronic submucosal inflammation. 

Our patient responded remarkably well to therapy and was afebrile on postoperative day 1. There was a small amount of reaccumulation of subgaleal fluid which was aspirated on two occasions and cultures remained negative. She was discharged home on intravenous Ampicillin/sulbactam through a peripherally inserted central catheter (PICC) and followed up in clinic. She continued to make progressive improvement. Twenty days into therapy, she developed a progressive, erythematous rash with itching unresponsive to diphenhydramine. In view of this development, antibiotic therapy was switched to intravenous meropenem via a peripheral inserted central catheter (PICC) in every 8-hour dosing. Her ESR remained stable at 27 mm/hr, and C-reactive protein had come down to 0.1 mg/dL. After 45 days of antibiotics, therapy was discontinued. She has continued to do well 2 years after therapy.

## 3. Discussion

In 1760, Sir Percival Pott first described Pott's puffy tumor as “a puffy, circumscribed, indolent tumour of the scalp and a spontaneous separation of the pericranium from the skull under such a tumour” [3]. Specifically, it is a frontal osteomyelitis with associated subperiosteal abscess of the frontal bone. In Pott's tumor, infection violates the anterior table of the frontal bone which leads to an abscess between the bone and periosteum—leading to its circumscribed appearance due to the adherence between periosteum and bone [4]. It is most commonly due to frontal sinusitis and may spread due to frontal sinus trauma, hematogenous spread of sinusitis, or retrograde thrombophlebitis via the diploic veins of Galen [5]. Pott's puffy tumor secondary to mastoiditis, insect bites, malignancy, and acupuncture has also been described [2, 6, 7]. 

Knowledge of the anatomy and embryology of the frontal sinus is critical in understanding the intracranial pathogenesis of Pott's tumor. The frontal sinuses develop from the ethmoid air cells and approach adult size between 12-13 years old [8]. Adolescence is the time when the vascularity of the diploic veins peaks [4]. Thus, intracranial involvement is possible without direct extension from the frontal bone since diploic veins are responsible for the venous drainage of the frontal sinus. With this understanding, it is logical that Pott's puffy tumor tends to be a complication of frontal sinusitis in older children—with a predilection for preteen and teenage boys [8]. 

Most commonly in Pott's puffy tumor, an untreated frontal sinusitis leads to an osteomyelitis of the anterior table of the frontal sinus and subperiosteal abscess formation. The anterior table of the frontal sinus is thinner than the posterior table and is more susceptible to abscess formation [5, 6]. As in this case, where there was involvement of the posterior table of the sinus, there is risk for intracranial involvement (epidural abscess, subdural empyema, meningitis, cavernous sinus thrombosis, cortical vein thrombosis, or sagittal sinus thrombosis) [1, 4, 6–8]. Moreover, involvement inferiorly can have orbital manifestations such as cellulitis or abscess. Presenting symptoms suggesting intracranial involvement include fever, seizures, headache, lethargy, vomiting, and focal neurologic deficits. Cases with intracranial abscess frequently manifest with headache [9]. Signs associated with increased intracranial pressure, nausea, vomiting, lethargy, and focal neurological signs are also commonly observed. As would be expected, most patients are presented either acutely or subacutely although development as late as 14 years after a traumatic head injury has been reported [9]. 

Although rare, given the potential for significant morbidity, a high index of suspicion for Pott's puffy tumor is required in patients presenting with fluctuant, tender, erythematous swelling of the scalp. Presentation can include headache, vomiting, fever, seizures, periorbital or scalp swelling, nasal or aural discharge, or focal deficits [2]. Signs and symptoms can range from a relatively indolent course consisting of headache, rhinorrhea, and fever to focal neurologic findings and altered consciousness [8]. Doughy swelling over the frontal area and low-grade temperatures are also frequently observed. Lab testing generally shows leukocytosis and an elevated ESR [2]. 

**Figure 4:**
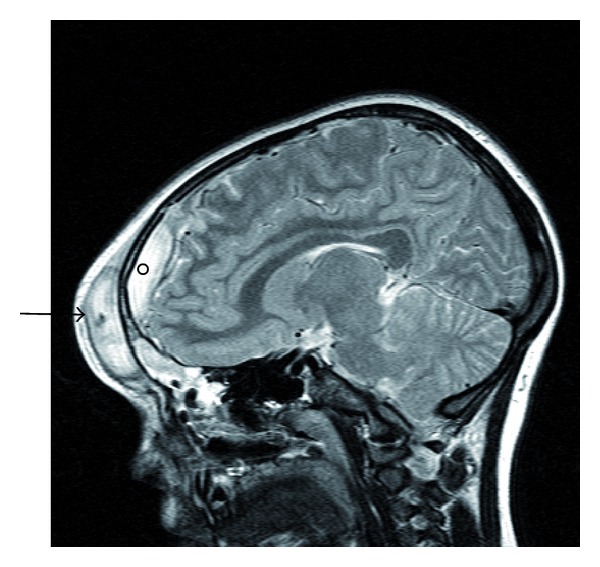
Sagittal MRI demonstrating an abscess within the frontal scalp (arrow) and an adjacent epidural abscess (circle).

CT of the brain is a diagnostic study of choice. It identifies intracranial and extracranial complications associated with frontal sinusitis [10, 11]. In this case, it showed extra- and intracranial fluid collections (Figures 2 and 3) consistent with Pott's puffy tumor. As in this case, MRI with gadolinium may help to further elucidate the extent of the disease intracranially [12] (Figure 4). 

Pott's puffy tumor is a surgical emergency. With the advent of modern functional endoscopic sinus surgery, this treatment can often be performed minimally invasively. In this case, treatment by both the otolaryngology and neurosurgery services was needed to address the concomitant sinus and intracranial disease. Frankly, diseased bone and granulation tissue require removal. Moreover, patency of the frontonasal duct must be reestablished [8]. Interventions such as an external frontoethmoidectomy, Caldwell-luc approach, and craniotomy are less frequently needed. Postoperatively, although the optimal time period is unclear, intravenous antibiotics for 6 to 8 weeks are generally accepted [5, 10, 12, 13]. Although not performed in this case, Maheshwar and colleagues [14] described the implantation of gentamicin beads to address the associated osteomyelitis. Review of the literature cannot identify this being used in other cases; however, its use in the orthopedic literature is well established. Intraoperative specimens are sent for microbiology in order to further direct antibiotic therapy.

Sinus cultures obtained intraoperatively are often polymicrobial. Organisms responsible for this disease process include alpha and beta hemolytic *Streptococci, Streptococcus pneumoniae, Staphylococcus, Haemophilus influenzae,* and less commonly *Proteus, Fusobacterium, Bacteroides,* and *Pseudomonas* [2, 6, 7]. These organisms may be more common in the setting of Pott's disease due to the low oxygen concentration in the obstructed frontal sinus [8]. In frontal sinusitis associated with intracranial complications anaerobes including *Fusobacterium, Bacteroides* species, and anaerobic, *Streptococci* are more frequent [12, 13]. 

Although initially described by Pott as a complication of trauma, this disease process is most frequent due to frontal sinusitis. As in this case, a high index of suspicion, accurate diagnosis, and prompt treatment are critical in achieving an optimal outcome.
